# Direct and up-close views of plant cell walls show a leading role for lignin-modifying enzymes on ensuing xylanases

**DOI:** 10.1186/s13068-014-0176-9

**Published:** 2014-12-31

**Authors:** Dragica Jeremic, Robyn E Goacher, Ruoyu Yan, Chithra Karunakaran, Emma R Master

**Affiliations:** Department of Sustainable Bioproducts, Mississippi State University, Starkville, MS 39759 USA; Department of Biochemistry, Chemistry and Physics, Niagara University, Lewiston, NY 14109 USA; Department of Chemical Engineering and Applied Chemistry, University of Toronto, 200 College Street, Toronto, ON M5S 3E5 Canada; Canadian Light Source Inc., 44 Innovation Boulevard, Saskatoon, SK S7N 2V3 Canada

**Keywords:** Lignocellulose, Cellulase, Xylanase, Laccase, Wood, Spectromicroscopy, Scanning transmission X-ray microscopy, Time-of-flight secondary ion mass spectrometry

## Abstract

**Background:**

A key barrier that limits the full potential of biological processes to create new, sustainable materials and fuels from plant fibre is limited enzyme accessibility to polysaccharides and lignin that characterize lignocellulose networks. Moreover, the heterogeneity of lignocellulosic substrates means that different enzyme combinations might be required for efficient transformation of different plant resources. Analytical techniques with high chemical sensitivity and spatial resolution that permit direct characterization of solid samples could help overcome these challenges by allowing direct visualization of enzyme action within plant fibre, thereby identify barriers to enzyme action.

**Results:**

In the current study, the high spatial resolution (about 30 nm) of scanning transmission X-ray microscopy (STXM), and the detection sensitivity (ppm) of time-of-flight secondary ion mass spectrometry (ToF-SIMS), were harnessed for the first time to investigate the progression of laccase, cellulase and xylanase activities through wood samples, and to evaluate complementary action between lignin-modifying and polysaccharide-degrading enzymes. In particular, complementary insights from the STXM and ToF-SIMS analyses revealed the key role of laccase in promoting xylanase activity throughout and between plant cell walls.

**Conclusions:**

The spatial resolution of STXM clearly revealed time-dependent progression and spatial distribution of laccase and xylanase activities, whereas ToF-SIMS analyses confirmed that laccase promoted protein penetration into fibre samples, leading to an overall increase in polysaccharide degradation. Spectromicroscopic visualizations of plant cell wall chemistry allowed simultaneous tracking of changes to lignin and polysaccharide contents, which provides new possibilities for investigating the complementary roles of lignin-modifying and carbohydrate-active enzymes.

**Electronic supplementary material:**

The online version of this article (doi:10.1186/s13068-014-0176-9) contains supplementary material, which is available to authorized users.

## Background

Cumulating genomic and metagenomic analyses underscore the critical role that microorganisms play in cycling the carbon fixed as lignocellulose in plant cell walls. Moreover, the vast collection of enzymes that have evolved to deconstruct plant biomass reflects the diversity of lignin and polysaccharide structures that comprise the main fraction of lignocellulose [1,2]. As succinctly summarized in a recent editorial overview [3], the discovery of new enzymes relevant to lignocellulose conversion to platform sugars for fuels and chemicals is enabled by our ability to predict polysaccharide substrate preference based on carbohydrate-active enzyme sequence. However, despite enormous strides in enzyme discovery and functional predictions, cost-effective bioconversion and utilization of lignocellulose remains a major challenge to fully realizing the potential of bioprocesses to partake in and inspire emerging bioeconomies based on renewable biomass sources.

The discrepancy between the discovery of lignocellulose-active enzymes and improved economics of lignocellulose processing is partly attributed to challenges encountered during functional expression of heterologous enzymes. However, once purified, many carbohydrate- and lignin-active enzymes are then characterized using model substrates that facilitate product detection but only partially reflect plant polysaccharide or lignin chemistry. By consequence, this approach does not capture the significance of composite lignocellulose architecture and heterogeneous distribution of lignocellulose components on enzyme efficiency (reviewed in [4] and [5]).

Advanced applications of complementary techniques including atomic force microscopy (AFM), confocal microscopy, infrared spectroscopy and mass spectrometry increasingly reveal cell wall features that affect enzyme performance [5-8]. For example, high-speed AFM provided the first real-time glimpse of cellulase action on cellulose fibrils at nanometre scales [9]. Also using AFM, Ganner *et al.* [10] subsequently collected mesoscopic views of cellulase action, and observed that synergism between endoglucanases and cellobiohydrolases is largely driven by the surface morphology of cellulose microfibrils. Whereas these studies evaluated the impact of cellulose structure on cellulase activity, Bayer and co-workers monitored the activity of fungal cellulases and cellulosome complexes on native and delignified corn stover [11]. By integrating several complementary imaging techniques including real-time visualizations of enzyme action, Bayer and co-workers observed that the detrimental effect of native lignin on cellulase activity largely results from the physical impedance of enzyme penetration through plant cell walls [11].

In addition to these techniques, mass spectrometry imaging (MSI) methods, including matrix-assisted laser desorption/ionization (MALDI-MSI), which spatially resolves sample chemistry to tens of micrometres, are becoming recognized as important techniques in plant biology [12]. A key benefit of MSI is the ability to determine the spatial distribution of specific molecules within a sample without the need for molecular tags or prior knowledge of sample chemistry. Similar to MALDI mass spectrometry, time-of-flight secondary ion mass spectrometry (ToF-SIMS) is a mass spectrometric technique used to determine the composition of a surface. In particular, by rastering a primary ion beam across the sample surface, it is possible to spatially map the surface chemistry to approximately 300 nm [13]. In an effort to develop effective screens of enzyme action on native and pretreated biomass, previous efforts by our group harnessed the detection sensitivity of ToF-SIMS (ppm) to characterize cellulase and laccase activity on ground wood fibre [14]. This procedure was recently optimized for robotic liquid handling and 96-well based enzyme screening [15].

Like ToF-SIMS and MALDI-MSI, scanning transmission X-ray microscopy (STXM) is a spectromicroscopic technique that can directly measure and image a sample’s chemistry. In STXM, a thin sample section (about 100 nm thick) is irradiated with finely focused soft X-rays of different energies, producing near edge X-ray absorption fine structure (NEXAFS) spectra characteristic of sample components. Although the detection limit of ToF-SIMS (ppm) surpasses that of STXM (%), STXM can be used to generate chemical maps with high spatial resolutions of about 30 nm [16]. To date, only a few studies have used STXM to analyse lignocellulose samples, including cell walls of tracheids from cedar and oak [17], composite materials comprising wood fibres and isocyanate resins [18], and kraft pulp before and after bleaching [19]. While overviewing spectral signatures for major organic molecules, Solomon *et al.* [20] further confirmed that reference spectra for aromatic compounds and carbohydrates are clearly distinguished.

The high spatial resolution of STXM and spectral sensitivity of ToF-SIMS have been harnessed here to visualize the effects of lignin-active and carbohydrate-active enzymes, alone or in combination, applied directly to wood fibre. Specifically, aspen wood sections were treated with laccase, xylanase and cellulase alone or as mixtures of laccase and xylanase. Wood sections were sampled over 10 days of enzyme treatment and analysed by STXM and ToF-SIMS. In this way, our aim was to study the progression of different enzyme activities on complex lignocellulosic substrates, and to visualize complementary activity between lignin-modifying and polysaccharide-degrading enzymes. To our knowledge, this is the first study that directly visualizes the effect of isolated lignin- and polysaccharide-active enzymes on the chemical distribution of lignocellulose components in wood fibre cell walls.

## Results and discussion

### Annotation of NEXAFS spectra

**Figure 1:**
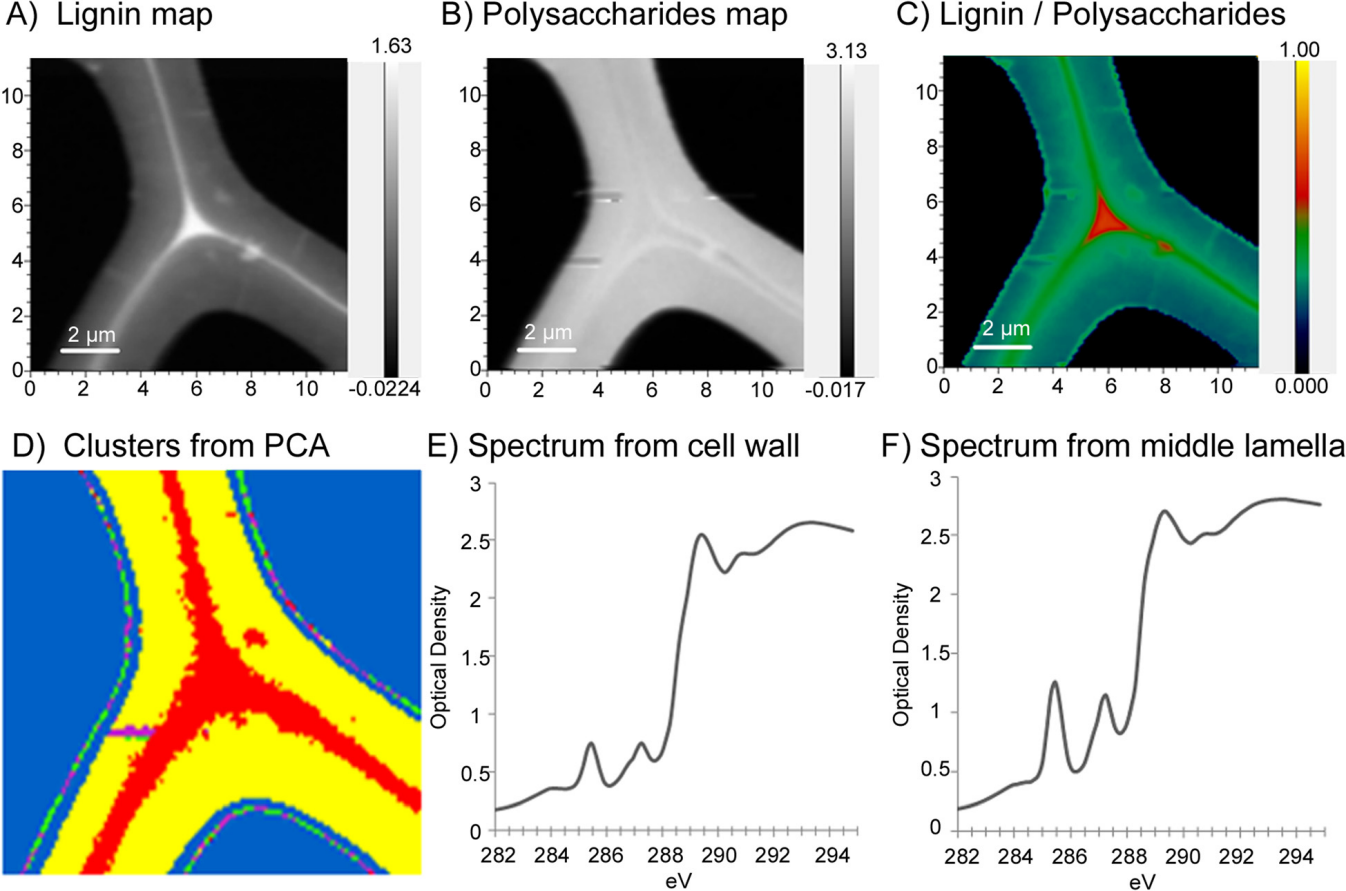
**STXM image processing steps. (A)** The lignin map calculated as the average of several energy images near the lignin peak at 287.3 eV, minus the pre-edge at 280.9 eV. **(B)** Polysaccharides map calculated using the average of several energy images near the polysaccharide peak at 289.3 eV, minus the pre-edge at 280.9 eV. **(C)** Map of lignin divided by polysaccharides (image **A** divided by image **B**) with false colouring (warmer colours indicate more lignin in a given region). **(D)** Map showing clusters determined by PCA-cluster analysis where pixels with same colours have similar composition; pixel colours are not correlated to colours used in lignin and polysaccharide maps. **(E)** Spectrum extracted from the yellow (cell wall) pixels in panel D. **(F)** Spectrum extracted from the red (middle lamella) pixels in panel **D**.

For STXM analyses, thin sample sections were scanned using focused X-rays at energies between 280 eV and 320 eV for carbon K-edge imaging and spectroscopy. Since there are relatively few published examples of lignocellulose samples that have been analysed using STXM, the interpretation of the resulting NEXAFS spectra was based on 1) previously published peak annotations (Additional file 1: Table S1), 2) preparation and analysis of reference samples (Additional file 2: Figure S1), and 3) known enrichment of lignin within the middle lamella region between wood cells (Figure 1). In all cases, the identification of characteristic peaks focused on the near edge absorption energy range of 282.0-292.0 eV.

The reference spectra clearly distinguished lignin and polysaccharides, with peaks in the lignin spectra close to 285.3 eV, 287.1 eV, and 288.5 eV, and peaks in the polysaccharide spectra near 289.3 eV along with a clear shoulder at 289.9-290.9 eV (Additional file 2: Figure S1). The NEXAFS spectrum of the 2,2′-azino-bis(3-ethylbenzothiazoline-6-sulphonic acid) (ABTS) mediator also showed characteristic absorbance for this molecule, with a shoulder at 286.0 eV, and peaks at 286.6 eV and 287.6 eV (Additional file 2: Figure S1). Conversely, besides a distinguishing feature in the shoulder near 287.5 eV, the Spurr’s resin, used to provide structural support to the microtomed sections, possessed spectra similar to that of protein samples, sharing absorption in the region of 285-285.6 eV and 288-288.8 eV. Notably, the similarity of protein and resin spectra makes it difficult to distinguish these components in wood sections and thereby specifically track enzyme penetration into the wood samples. Nevertheless, one can reasonably infer that the occurrence of a peak near 288.5 eV in spectra from middle lamella or cell wall regions, be it from protein and/or resin, would likely result from increased substrate accessibility resulting from enzyme action.

The aspen control spectrum (shown in Additional file 2: Figure S1) demonstrates a slight shift in peaks for cellulose and lignin compared to the isolated standards. Since the energy scale was routinely calibrated to CO_2_, this shift was not due to a drift in beamline energy. Instead, peak maxima for a given component can shift slightly depending on the presence of other compounds. Accordingly, an initial analysis of STXM images was performed to validate peak energy assignments for lignin and polysaccharides present in aspen wood fibre. Specifically, a map of lignin (Figure 1A) and polysaccharides (Figure 1B) was obtained for an untreated wood cross section as the sum of images at energies characteristic of lignin (near 287.3 eV) and polysaccharides (near 289.3 eV), respectively. Resulting maps were then superimposed by calculating the ratio of intensities of the selected lignin and polysaccharide peaks (Figure 1C).

As expected, comparatively high lignin intensities were observed in the middle lamella region between neighbouring cell walls, with the highest concentrations in cell corners, whereas polysaccharide intensities were highest in the secondary cell wall region. The consistency between the interpretation of NEXAFS spectra and prior knowledge of wood cell wall chemistry confirms the applicability of published and reference peak assignments to the current lignocellulose samples (Additional file 1: Table S1). Nevertheless, to further validate subtle shifts in peak maxima for lignin and polysaccharide components, and to check if other prominent peaks of reference spectra could be used for differentiation, PCA-cluster analysis was performed over the spectral range between 282 to 295 eV. The obtained clusters readily resembled the middle lamellae and cell wall regions of neighbouring fibres (Figure 1D), and spectra representing each cluster revealed patterns with higher intensities at 285.3 and 287.1 eV for lignin-rich regions and higher intensities at 289.3 eV for polysaccharide-rich regions (Figure 1E-F). Furthermore, these peak annotations were repeatedly confirmed through differences in middle lamella and cell wall spectra observed in PCA models of the STXM data, as discussed below. Given the closeness in eV values reported in earlier publications (Additional file 1: Table S1), measured from reference spectra (Additional file 2: Figure S1), and identified through PCA clusters of NEXAFS spectral images from middle lamellae and cell wall regions (Figure 1), the peak energies summarized in Additional file 1: Table S1 are reported in the sections below for simplicity, and were within the nominal precision of 0.15 eV of measured values.

### STXM analysis of cellulase, xylanase and laccase treated wood samples

The first set of STXM experiments was designed to explore whether enzyme action on wood could be directly visualized using STXM. Accordingly, enzymes that targeted lignin and main cell wall polysaccharides were included in the analysis. Since xylanase activity can also promote the release of lignin (reviewed in [21]), initial analyses also included aspen wood samples treated with both xylanase and laccase.

Similar to the map shown in Figure 1C, maps of lignin-to-polysaccharide peak ratios using peaks centred at 287.1 eV (lignin) and at 289.3 eV (polysaccharides) differentiated the middle lamella and cell wall regions of control and enzyme treated samples, where the greatest intensity at 287.1 eV was observed in the cell corners and middle lamella (Additional file 3: Figure S2 A-F). Pixels within each individual image stack were then grouped by PCA-cluster analysis as shown in Figure 1D, and corresponding spectra for each cluster (as shown in Figure 1E-F) were compared by PCA to identify changes in cell wall and middle lamella chemistry across all of the samples. The differences in middle lamella and cell wall spectra from all of the samples confirmed peak annotations identified by the PCA-cluster analysis of STXM images.

**Figure 2:**
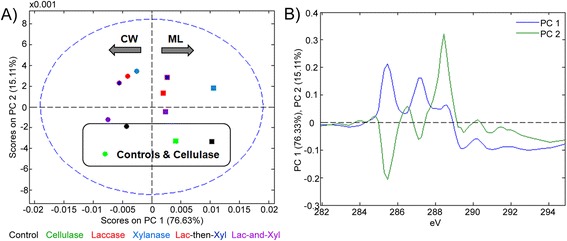
**Principal component analysis (PCA) of initial NEXAFS spectra.** PCA scores **(A)** and loadings **(B)** for NEXAFS spectra extracted from the cell walls (CW, circles) and middle lamellae (ML, squares) for control, cellulase and different xylanase:laccase co-treatments. Outlines on scores plots are to guide the eye.

Consistent with the maps of lignin-to-polysaccharide peak ratios, PC1 of this analysis clearly described the enrichment of lignin in the middle lamella versus enrichment of polysaccharides in cell wall regions (Figure 2A-B). More specifically, the middle lamellae of all samples were distinguished by lignin peaks near 285.3 eV and 287.1 eV, and the cell walls of all samples were distinguished by polysaccharide peaks near 289.3 and 291.0 eV. While data replicates were limited for this analysis, the middle lamellae of the control and xylanase treated samples appeared more lignin-rich, scoring more positively on PC1, with the lignin-related peaks.

The impact of enzyme treatments on cell wall chemistry was further explained by PC2, where samples that scored negative (cellulase treated and control samples) were lignin-rich as indicated by the peaks near 285.3 and 287.1 eV (Figure 2A-B). Samples that scored positive on PC2 (xylanase, laccase, and laccase-then-xylanase treatments) were depleted in the main lignin peaks and were instead enriched in a peak near 288.5 eV characteristic of Spurr’s resin as well as protein (Figure 2A-B), indicating more extensive penetration of the embedded resin and/or applied protein as a result of enzyme action.

It is conceivable that the higher impact of the two-day treatment with laccase and xylanase on cell wall chemistry compared to cellulase reflects the comparatively low molecular weight of the xylanase used herein and the ABTS mediator of laccase activity. Others have shown that micropore size in untreated and pretreated wood samples is polydisperse and can vary between 10-800 Å, even though an average micropore size between 30-40 Å is typically reported (reviewed in [22]). The polydispersity of micropores in lignocellulosic materials leads to both slow and fast diffusion coefficients for single enzymes and molecular probes [23,24]. For example, polyethylene glycol (PEG) with a radius of gyration of approximately 30 Å was shown to readily penetrate wood cell walls [25,26], whereas dextran probes with hydrodynamic molecular diameters between 40 to 90 Å penetrate micropores of filter paper, albeit at different rates [24]. By comparison, the dimensions of a glycoside hydrolase (GH) family 11 xylanase from *Thermomyces lanuginosus* is reported to be between 40 Å × 38 Å × 35 Å [27], and the corresponding value for the GH7 endoglucanase from *Trichoderma reesei* is 60 Å × 50 Å × 40 Å [28]. In this case, it is likely that the xylanase as well as the ABTS mediator (514.62 Da) would migrate further through the micropores present in wood than the cellulase mixture used in these experiments.

The comparatively low impact of cellulase treatments likely also reflects poor accessibility to the comparatively crystalline cellulose substrate [29]. Real-time microscopic analyses of cellulase action on corn stover demonstrate that accessibility to cellulose is restricted in part by the dense lignin barrier, or “warty layer”, of secondary cell walls [11]. It is also becoming increasing clear that co-hydrolysis of xylan during cellulose hydrolysis promotes swelling of cellulose microfibrils, which can increase the access of cellulose to applied cellulases [30]. The current analyses similarly suggest that breaking through lignin and hemicellulose networks, either through laccase-mediated modification of lignin or xylanase-mediated solubilization of xylan and associate lignin structures, is critical to the further penetration of applied enzymes through plant cell walls.

### STXM analysis of xylanase and laccase co-treated wood samples

The potentially complementary action of xylanase and laccase on the progression of enzyme activity through lignified wood samples motivated additional STXM analyses of laccase and xylanase treatments, this time altering enzyme doses and incubation times. To evaluate the impact of laccase on xylanase activity and vice versa, five enzyme mixtures were compared with comparatively high, low, or equal doses of each enzyme (9:1, 3:1, 1:1, 1:3 and 1:9 mixing ratios by mass). Since time for STXM analyses was limited, and the analysis of multiple replicates was prioritized higher than analysis of more sample types, the ToF-SIMS analysis discussed below was used to limit the samples for STXM analysis to the 9:1, 1:1, and 1:9 treatments. Furthermore, since it was predicted that the complementary action of these enzymes might change over time as a result of lignin and polysaccharide degradation, three time points (1 day, 5 days and 10 days) were also included in the study.

As observed in single enzyme treatments, the lignin-to-polysaccharide maps of all co-treatments clearly delineated middle lamella and cell wall regions, and revealed comparatively high lignin content in the middle lamella and cell corners of control samples (Additional file 4: Figure S3). Notably, in addition to differentiating middle lamella and cell wall regions using lignin-to-polysaccharide maps, PCA-cluster analysis further distinguished S1 and S3 cell wall layers from the S2 layer in all samples treated for 10 days as well as all samples treated with high xylanase dose (Additional file 5: Figure S4, Additional file 6: Figure S5).

**Figure 3:**
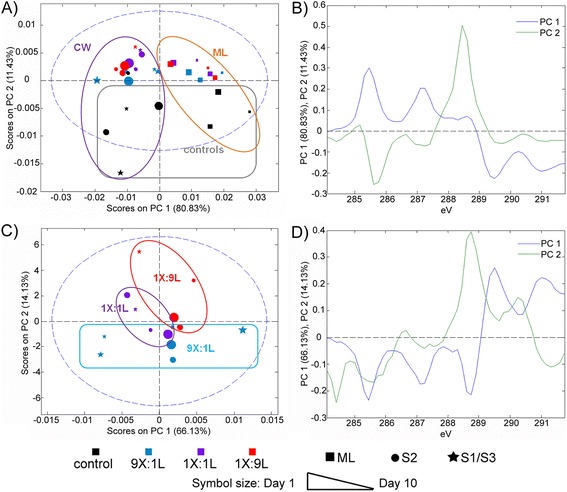
**PCA of aspen samples before and after treatment with xylanase and laccase mixtures.** PCA scores **(A, C)** and loadings **(B, D)** comparing STXM clusters from middle lamellae (ML) and cell walls (CW) for different xylanase:laccase co-treatments. PCA models compare all samples and all cell regions **(A, B)**, and only enzyme-treated cell wall layers (both S1/S3 and S2) **(C, D)**. Outlines on scores plots are to guide the eye.

PCA scores and loadings of these cluster spectra were analysed to objectively reveal changes to the spatial distribution of sample chemistry across enzyme treatments (Figure 3; Additional file 5: Figure S4). First, all sample regions for all enzyme treatments were analysed together with controls (Figure 3A-B). As previously observed, PC1 distinguished middle lamella and cell wall regions of all samples by their relative enrichment in lignin peaks near 285.3 eV and 287.1 eV and polysaccharide peaks near 289.3 eV and 291.0 eV, respectively (Figure 3A-B). PC2 distinguished all enzyme treated samples from the control samples where enzyme treatments were characterized by a peak near 288.5 eV in both middle lamella and cell wall spectra (Figure 3B). This result was consistent with earlier analyses using laccase and xylanase separately and sequentially, where again the peak near 288.5 eV likely reflects increased protein and/or resin penetration into the wood sample as a result of enzyme action.

To further explore compositional differences between S1/S3 cell wall edges and the S2 inner cell wall layer, PCA was performed on spectra of clusters describing cell walls of enzyme-treated samples without including middle lamella regions or control samples (Figure 3C-D; Additional file 5: Figure S4). In this analysis, PC1 revealed a time-dependent depletion in polysaccharides within S1/S3 layers of samples treated with high xylanase doses (Figure 3C-D, blue star symbols). More specifically, the corresponding loadings distinguished spectra of day-1 samples by peaks characteristic of polysaccharides near 289.3 eV and 291.0 eV and spectra of day-5 and day-10 samples by peaks characteristic of lignin near 285.3 eV and 287.1 eV as well as resin near 288.5 eV. This observation suggests that even in samples treated with a comparatively high xylanase dose, the progression of polysaccharide degradation followed initial lignin degradation, causing a transition from polysaccharide-enriched cell wall edges to lignin-enriched cell wall edges only after prolonged incubation times. Similarly, after 10 days of incubation, PCA-cluster analysis distinguished the S1/S3 layers of fibre cell walls treated with equal doses of xylanase and laccase, and high laccase doses (Additional file 5: Figure S4). In particular, the corresponding spectra for high-laccase treatments revealed high protein and/or resin as well as lignin contents in these cell wall regions compared to the S2 layers (Figure 3C-D, PC1, red star symbols). This would again indicate initial lignin degradation across the cell walls at early time points, and eventual polysaccharide depletion at cell wall edges as xylanase gains further access to the xylan substrate.

PC2 of cell wall spectra from enzyme treated samples mainly differentiated high xylanase and high laccase treatments (Figure 3C-D). Whereas samples treated with low laccase doses had no distinguishing and annotated peaks in their PC2 loadings, the spectra of cell walls from high laccase treatments were characterized by the resin peak near 288.5 eV as well as by a peak at 290.3 (polysaccharides). The higher contribution of the peak near 288.5 eV in spectra from samples treated with high laccase doses suggests that the activity of laccase is particularly important in increasing sample accessibility.

PCA was also performed on the spectra of clusters describing middle lamella regions of enzyme treated samples without including cell wall regions or control samples (Additional file 7: Figure S6). In this case, PC1 distinguished treatments according to incubation time, and while PC1 and PC2 cumulatively accounted for nearly 90% of the variance within this dataset, samples were not grouped by enzyme treatment (Additional file 7: Figure S6 A). Inspection of loadings for PC1 revealed that similar to the results for cell wall edges, all enzyme treatments led to middle lamella regions that were initially enriched in polysaccharides (with a peak near 289.3 eV) and then enriched in lignin only after prolonged incubation (with peaks near 285.3 eV and 287.1 eV), again suggesting that initial lignin degradation was required to promote subsequent polysaccharide degradation in middle lamella regions as well as fibre cell walls (Additional file 7: Figure S6 B).

### ToF-SIMS analysis of unembedded wood samples

While STXM offers exquisite spatial resolution of sample chemistry, the spectral sensitivity of ToF-SIMS provides a complementary method to directly characterize small changes in sample chemistry. The comparative speed of data collection by ToF-SIMS also meant that multiple samples could be analysed to confirm the biological reproducibility of the compositional changes observed using STXM. In particular, ToF-SIMS was used in the present study to further examine aspen wood samples treated for up to 10 days with varying ratios of laccase and xylanase doses. Wood fibres were sampled from the same reaction mixture that generated samples for STXM analyses. However, rather than solvent-exchange drying and embedding the sample, wood fibres were air-dried and then ground to powder to detect overall changes to chemical composition.

Earlier work in our lab expanded upon peak annotations that distinguish lignin and polysaccharides in ToF-SIMS spectra from wood samples [31]. This list was later refined to identify unique peaks and peak interferences resulting from applied enzymes [14]. Most recently, multivariate curve resolution (MCR) was used to reveal pure component spectra of residual buffer salts and adhesive tape in complex ToF-SIMS spectra from enzyme treated wood samples [15,32].

**Figure 4:**
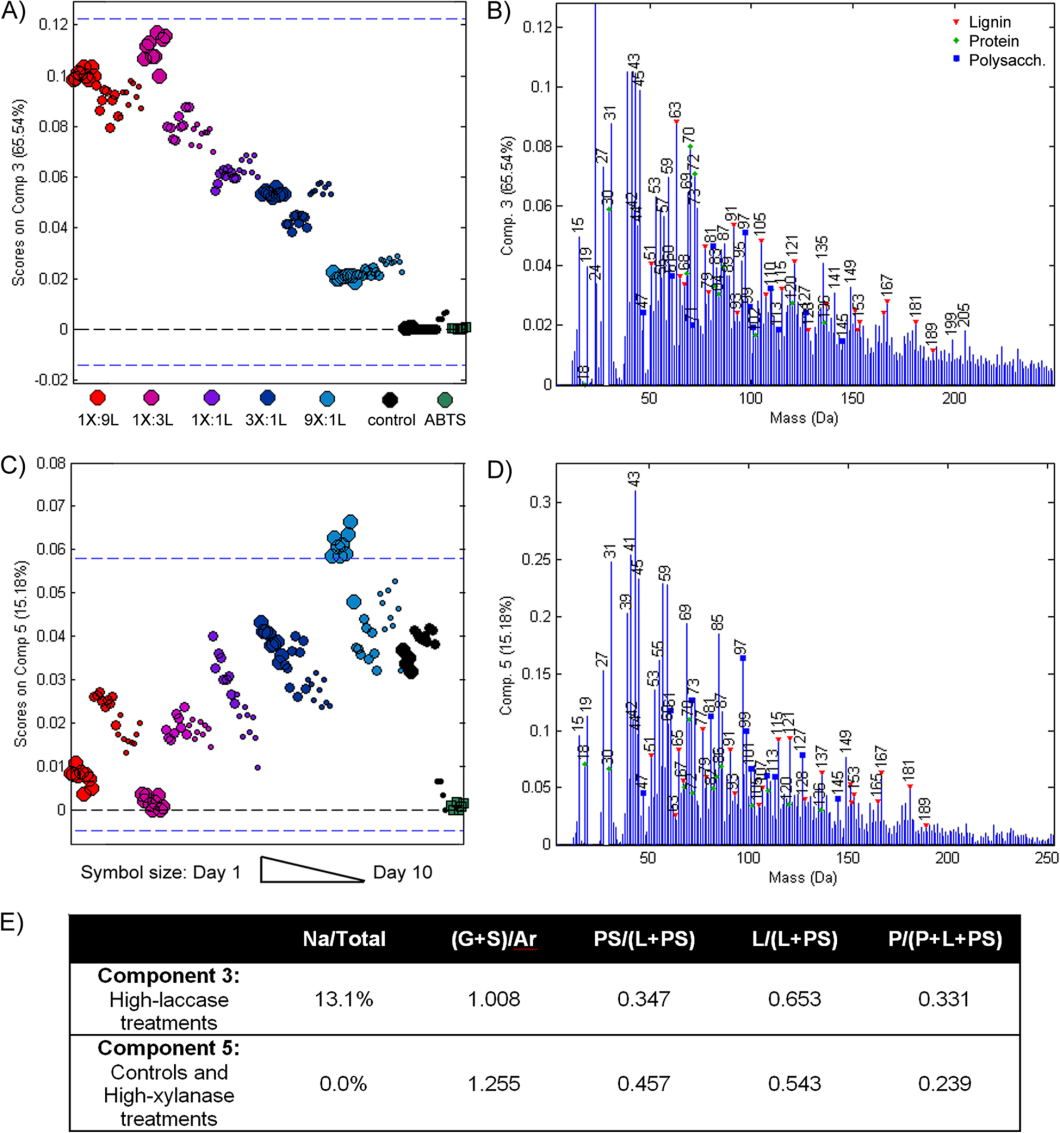
**MCR analysis of ToF-SIMS spectra.** Scores **(A, C)** and corresponding component spectra **(B, D)** for the two components related to the main wood biopolymers. Comparison of **(A)** and **(C)** illustrate changes due to the xylanase:laccase mixing ratio. Panel **E** tabulates five descriptive peak ratios from the pure component spectra. These ratios are: Na^+^ (23 Da) as a percentage of the total counts in the component spectrum; lignin modification metric describing ions characteristic of guaiacyl (G) and syringyl (S) lignin relative to generic aromatic (Ar) ions; polysaccharide peak fraction describing the proportion of total lignin (L) and polysaccharide (PS) peaks; and protein coverage (P) as previously reported [13,15].

Although mass overlap in ToF-SIMS between ABTS and lignin fragment ions was not observed in prior work using 1 mM ABTS, the greatly increased ABTS concentration of 50 mM used herein merited MCR analysis of ToF-SIMS spectra for all wood sample spectra as well as pure ABTS spotted onto a silicon wafer. In this analysis, ABTS contributions to overlapping peaks were detected and described by MCR component 1 (Additional file 8: Figure S7 A-B), leaving wood-related ions in other components free of interferences from ABTS. Since samples were prepared in glass scintillation vials to avoid contamination from plasticizers (such as polydimethyl siloxane, PDMS), most samples were free from PDMS contamination. Nevertheless, the second MCR component described PDMS peaks in a few wood spectra (Additional file 8: Figure S7 C-D), likely introduced when grinding the samples for ToF-SIMS analysis. Finally, as observed by Goacher *et al.* [15], the MCR model also detected spectra arising from the adhesive tape used to immobilize the wood powder to a glass slide for ToF-SIMS analysis (Additional file 8: Figure S7 G-H). Consequently, for purposes of comparing chemical changes due to enzymatic degradation, the most informative MCR components were components 3 and 5, which described wood samples treated with a high laccase dose and a high xylanase dose, respectively (Figure 4, Additional file 8: Figure S7 E-F, I-J).

Overall, the trends revealed by calculated peak ratios from the original ToF-SIMS spectra (Additional file 9: Figure S8) were in agreement with the MCR model (Figure 4). Component 3 of the MCR model, as well as the original spectra ratios, revealed significant levels of Na^+^ ions (23 Da), particularly in wood samples treated with more laccase (Figure 4A-B, Additional file 9: Figure S8 A). ToF-SIMS analysis of stock laccase and xylanase solutions showed nearly equal amounts of Na^+^ ions. Accordingly, higher Na^+^ ion intensity in samples treated with increasing laccase dose could be due to increased penetration of enzyme solutions into corresponding wood samples. The spectra from components 3 and 5 of the MCR model, along with the original spectra, were also used to compare polysaccharide, lignin and protein content in enzyme treated and control samples using previously defined peak ratios [14,15] and excluding the salt ion interferences noted in Goacher *et al.* [15] with lignin at 63 and 165 Da (Additional file 9: Figure S8 B-D). In addition to higher Na^+^ ion content, samples treated with a high laccase dose had higher protein content compared to untreated samples and samples treated with a high xylanase dose (Figure 4, Additional file 9: Figure S8). Considering the detection of the resin- or protein-related peak near 288.5 eV in the NEXAFS spectra of samples treated with high laccase doses, the comparatively high Na^+^ ion and protein content observed in corresponding samples analysed by ToF-SIMS provides further evidence that laccase promoted both protein and resin penetration through the wood samples.

Interestingly, the relative intensities of polysaccharide peaks were lower in samples treated with high laccase doses compared to those treated with high xylanase doses, indicating higher polysaccharide loss from treatments subject to high laccase rather than high xylanase activity (Figure 4B and D, Additional file 9: Figure S8 C). At the same time, the proportion of G-lignin and S-lignin monomer peaks (137, 151, 167 and 181 Da) relative to the generalized aromatic peaks (77 and 91 Da) were also lower in high laccase treatments (Figure 4). A decrease in this (G + S)/Ar ratio was previously linked to lignin modification by the laccase-ABTS system [14]; mass spectrometric analysis of reaction supernatants performed herein also suggested that ABTS had lowest concentrations in reactions containing a comparatively high laccase dose (Additional file 10: Table S2). Taken together, the lower intensity of polysaccharide peaks and lower (G + S)/Ar ratio in samples treated with a high laccase dose further supports the idea that laccase activity promotes both ABTS and xylanase penetration into plant cell walls, and increases xylanase access to the xylan substrate.

## Conclusions

The spatial resolution and spectral sensitivity of STXM and ToF-SIMS, respectively, allowed the direct visualization of complementary laccase and xylanase activities. Whereas the spatial resolution of STXM clearly revealed time-dependent as well as spatial progression of lignin-modifying and subsequent polysaccharide-degrading activities, ToF-SIMS analyses confirmed that samples treated with a high laccase dose promoted protein penetration into fibre samples, leading to an overall increase in polysaccharide degradation. In particular, while STXM analysis revealed more extensive loss of polysaccharides from the cell wall edges of wood samples treated with a high xylanase dose, ToF-SIMS analysis of bulk compositions showed that polysaccharide content was reduced in all samples, and that the highest overall reductions were observed in high laccase treatments.

The current study suggests a key role for lignin-modifying enzymes, including laccases, in promoting xylanase activity within and between plant cell walls. There is also increasing evidence that the action of xylanases subsequently increases access of cellulases to cellulose [30]. The role of xylanases in increasing cellulose accessibility is further supported by modelling studies that predict contributions from different hemicellulose types on maintaining the integrity of cellulose microfibrils and effectively increasing biomass recalcitrance to enzymatic processing [33]. The development and optimization of sample preparation and data analysis presented herein will facilitate applications of STXM and ToF-SIMS designed to characterize complementary and possible synergistic action between lignin- and polysaccharide-active enzymes. In particular, through direct visualizations of plant cell wall chemistry and the ability to simultaneously track changes in lignin and polysaccharide contents, future applications of STXM and ToF-SIMS are ideally suited to evaluate the impact of different lignin-active enzymes, as well as laccase mediators, on the action of glycoside hydrolases in general, and the ensuing role of hemicellulases in increasing cellulose accessibility.

## Methods

### Wood sample preparation

Wood sections of aspen (*Populus tremuloides*) approximately 3 × 3 × 10 mm^3^ (tangential × radial × longitudinal directions) were prepared from a single annual ring and end-matched to minimize initial chemical variability between samples. Notably, the sample length (10 mm) was chosen to minimize possible obstruction of applied enzymes by the presence of bordered pits between adjacent fibres. Wood sections were Soxhlet extracted in an azeotropic mixture of ethanol:toluene (1:0.427 v/v) for 4 h, followed by ethanol for 8 h, according to ASTM standard D 1105. Extractive-free samples were then air-dried at room temperature before use.

### Enzyme treatments

Commercial enzymes were chosen to catalyse lignin depolymerization (Novozym 51003; laccase from *Myceliophthora thermophila*), xylan hydrolysis (Novozym 51024; GH11 xylanase from *Thermomyces lanuginosus*), and cellulose hydrolysis (Novozym Celluclast 1.5 L). Two sets of experiments were then performed, and in both cases, reactions were conducted in glass vials containing five solvent extracted wood sections and 3 mL of enzyme solution prepared in de-ionized (MilliQ) water (the final pH was approximately 5). The first set of experiments compared the impact of single enzyme treatments to treatments containing both hydrolytic and oxidative enzymes. In these experiments, single enzyme treatments consisted of aspen wood sections treated for two days at 50°C with 1 mg/mL of the cellulase mixture, 5 mg/mL xylanase, or 1 mg/mL laccases and 10 mM 2,2′-azino-bis(3-ethylbenzothiazoline-6-sulphonic acid) (ABTS, Sigma Aldrich); cellulase and xylanase loadings were adjusted to comparable units of hydrolase activity. Co-enzyme reactions included simultaneous treatment of aspen sections with 5 mg/mL xylanase, 1 mg/mL laccase, and 10 mM ABTS for two days at 50°C, as well as corresponding sequential treatments at 50°C for a total of four days (two-day incubations for each enzyme).

The second set of experiments compared the impact of varying enzyme dose and incubation time. In these experiments, a minimum of five aspen wood sections were treated with varying doses of xylanase and laccase. The relative abundances of xylanase and laccase in the reaction mixtures were adjusted to 1:9, 1:3, 1:1, 3:1 or 9:1 by weight, giving a final protein concentration of 5 mg/mL; reactions were also amended with 50 mM ABTS. High protein concentrations were used to provide a driving force for the diffusion of proteins into wood pores. Maintaining the same total enzyme dose on a milligram basis also addressed the possibility that enzyme activities can be enhanced simply through having more total protein in a reaction mixture. Furthermore, this experimental set-up allowed semi-quantitative interpretation of NEXAFS and ToF-SIMS spectra, which describe specific elements and molecular fragments, respectively, rather than moles of particular molecules (for example, proteins) [5]. Reaction solutions were replaced with fresh enzyme and ABTS on a daily basis. Triplicate vials were prepared, with the treatment ended and wood sections sampled after 1 day, 5 days and 10 days of incubation at 50°C.

Control samples were prepared by incubating wood sections in MilliQ water alone or with ABTS, and the concentration of enzyme stock solutions was determined using the Bradford assay. Laccase action on ABTS at pH 5.0, as well as xylanase activity in the presence of 50 mM ABTS, was confirmed using standard colourimetric assays [34,35]. Laccase and xylanase activity in reactions containing wood samples was also confirmed by qualitative detection of solubilized xylo-oligosaccharides and consumption of ABTS in reaction supernatants using mass spectrometry (Additional file 10: Table S2).

### Thin section preparation for STXM analyses

To assess changes in wood fibre chemistry that resulted from enzyme penetration through the wood sample, the core 1 × 1 × 5 mm^3^ regions of enzyme treated wood sections were recovered and then solvent exchange-dried using increasing concentrations of ethanol (30 min in 20%, 40%, 60%, and 95% ethanol, followed by 3 × 30 min in 100% ethanol). The wood sections were then embedded under vacuum using increasing concentrations of low viscosity Spurr’s resin (Canemco-Marivac; 30 min each in 50% and 67% Spurr’s resin in ethanol, followed by 4 h and 18 h in fresh preparations of 100% Spurr’s resin). The resin embedded samples were polymerized at 60°C for 16 h, and after curing sections approximately 100 nm thick were prepared using a Leica EM UC6 ultramicrotome equipped with a DiAtome Ultra diamond knife. The sections were mounted on 200 mesh uncoated copper grids (SPI Supplies). The reference samples, including 2 μL of a 50 mM ABTS solution, were air-dried directly on silicon nitride windows (SPI Supplies).

### STXM analyses

Scanning transmission X-ray microscopy (STXM) measurements were made at the C 1 s edge using the STXM end station on the spectromicroscopy beamline of the Canadian Light Source (CLS). A Fresnel zone plate that can focus the X-ray beam to 25 nm was installed for these measurements. Circular polarization of the X-rays was used to reduce orientation affects from sample components, including cellulose, and the titanium filter was used to remove any second order light within the X-ray beam. The energy scale of the beamline was calibrated using CO_2_ absorption as a reference (294.96 eV). Image stacks were collected in transmission mode under vacuum conditions by scanning the sample in X and Y directions at each individual beam energy. The beam energy was changed in increments of 0.5 eV from 280.0 to 284.0 eV; 0.15 eV from 284.2 to 292.0 eV; 0.392 eV from 292.2 to 302.0 eV; and 0.978 eV from 302.4 to 320.0 eV, with a 1-ms dwell per pixel or spot size. The smaller energy step (0.15 eV) was used in regions of the NEXAFS spectrum where core-shell C 1 s electron excitations occur, and this region of the spectrum was the main focus of subsequent PCA-cluster analysis. The dimensions of the imaged areas were chosen to visualize the intersection of three to four fibres, to thereby clearly observe cell wall and middle lamella regions at high spatial resolution and at a reasonable data acquisition time. The pixel size of the image stacks used in these analyses was 100 nm.

The NEXAFS spectra were analysed using aXis 2000 software (http://unicorn.mcmaster.ca/aXis2000.html), and were converted from transmission to absorption (optical density) using the I0 spectra from empty spaces in the lumen. Upon manual examination of each stack, images that showed uneven intensities at some lines across the scanned areas (due to beam instabilities) were corrected using the median-filter function and then included with the stack. Stacks were subsequently aligned using a constant image which had the highest contrast or using an image with edges enhanced using the Sobel procedure. It was observed that in some regions of the wood samples, the embedding resin folded and moved with increased X-ray exposure, which affected subsequent statistical analysis of STXM images. Thus, stacks were averaged and multiplied by a mask of background images to remove the resin regions. The aXis 2000 software was then used to perform PCA-cluster analysis of the aligned and energy calibrated stacks [36-38]. In this way, the number of clusters to include in the subsequent PCA analysis of corresponding cluster spectra was objectively determined.

### ToF-SIMS analyses

Unembedded wood sections were also analysed by time-of-flight secondary ion mass spectrometry (ToF-SIMS). In this case, the wood samples were not resin embedded but were directly ground into a fine powder using a bead beater with stainless steel beads and vials with polyethylene stoppers. The powdered wood samples were then pressed onto double-sided Scotch® adhesive tape supported by a glass slide.

ToF-SIMS measurements were performed using a ToF-SIMS IV instrument (ION-TOF GmbH, Münster, Germany) equipped with a bismuth liquid metal ion source and reflectron-type analyser with multichannel detector. Positive ion spectra were acquired using 50 keV Bi_3_^2+^ primary ions (about a 0.3-pA pulsed current) incident at 45°, operated on a 100-μs cycle time with high-current bunched conditions. The instrument’s stage-scanning abilities were used to scan each sample over a 2000 × 2000 μm^2^ area with a 200 × 200 pixel raster pattern. One hundred primary ion shots were used per pixel, keeping the primary ion dose density below 1 × 10^−11^ ions/cm^2^ to limit sample damage. Each scan was sub-divided into nine regions of interest to assess reproducibility within each wood sample. The pressure during analysis was maintained below 4 × 10^-7^ mbar. Low energy electron flooding (20 eV) was used to reduce sample charging. Positive ion spectra were calibrated to CH_3_^+^, C_2_H_3_^+^ and C_3_H_5_^+^ ions using SurfaceLab 6.3 software (ION-TOF GmbH, Münster, Germany). The mass resolution (M/ΔM) varied depending on sample roughness, and all the ToF-SIMS data were binned to 1 Da before calculating peak ratios or applying statistical analysis.

### Statistical analyses

Principal component analysis (PCA) and multivariate curve resolution (MCR) of NEXAFS cluster spectra and ToF-SIMS spectra were performed using Matlab software v.8.0 (The Mathworks, Inc.) with PLS Toolbox version 7.0.3 (Eigenvector Research, Inc.). ToF-SIMS spectra were processed using Poisson scaling (square root mean scaling) and unit normalization, and PCA was used to determine the appropriate number of curves for MCR. NEXAFS cluster spectra in the range of 281.9 - 291.75 eV were normalized to total intensity (sample thickness) prior to PCA. Effects of time and enzyme mixture on ToF-SIMS data were examined statistically through one-way ANOVA and Tukey-Kramer analysis using SAS software (SAS Institute).

## Additional files

Additional file 1: Table S1. Characteristic NEXAFS peaks for ABTS, lignin, polysaccharides, protein and resin [39-42]. Additional file 2: Figure S1. Reference NEXAFS spectra. NEXAFS spectra of protein (bovine serum albumin), Spurr’s resin, xylan, cellulose, lignin, ABTS, and 1-day control aspen over the near-edge energy range of 282.0-292.0 eV are shown. Spectra for ABTS, Spurr’s resin, and aspen were collected in this current study; other spectra were previously collected by the Canadian Light Source (CLS). Additional file 3: Figure S2. STXM ratio images showing lignin to polysaccharides (as in Figure 1C) for untreated aspen (A), and for aspen treated with cellulase alone (B), laccase alone (C), xylanase alone (D), laccase followed by xylanase (E) and laccase co-incubated with xylanase (F). Additional file 4: Figure S3. STXM images showing lignin maps at 287.1 eV divided by polysaccharide maps at 289.3 eV for aspen treated with varying doses of xylanase and laccase for 1, 5, and 10 days. Images were prepared in the same way as Figure 1C. All images use the same colour scale, with warmer colours indicating more lignin-rich regions and cooler colours indicating more polysaccharide-rich regions. Additional file 5: Figure S4. Results of PCA-cluster analysis of STXM maps for samples treated with various doses of xylanase and laccase mixtures and ABTS for 1 day (top), 5 days (middle) and 10 days (bottom). Control samples were treated with ABTS only. The same colours within each image represent areas with the same chemistry. However, the colours between different images do not necessarily imply the same chemistry. Additional file 6: Figure S5. Clusters within STXM maps showing S1/S3 layers (top, green) and S2 layers (bottom, yellow) for samples treated with high doses of xylanase (9X:1 L) for different periods of time (left: 1 day, middle: 5 days, right: 10 days). Additional file 7: Figure S6. PCA scores (A) and loadings (B) comparing STXM clusters from enzyme-treated middle lamella of the samples treated with varying xylanase and laccase ratios. Additional file 8: Figure S7. MCR analysis of ToF-SIMS spectra showing scores (A, C, E, G, I) on the five component spectra (B, D, F, H, J). Component 1 (A, B) describes the ABTS mediator. Component 2 describes poly (dimethyl siloxane) (PDMS) contamination. Component 3 describes Na, protein, and lignin-rich wood. Component 4 describes the tape used to support the powdered wood. Component 5 describes lignin-depleted wood. Additional file 9: Figure S8. Peak ratios from the raw ToF-SIMS spectra of the ground aspen samples, as described in Sodhi [13]: A) the proportion of the total spectrum comprised by the Na + peak at 23 Da; B) the lignin modification metric; C) the polysaccharide peak fraction and D) protein coverage. Peak ratios of pure ABTS deposited on a silicon wafer are also shown, to indicate whether ABTS coverage would increase or decrease the peak ratio. Notably, while Na + ions alone do not directly interfere with lignocellulose and protein peaks, at high levels of Na+, salt cluster ions have been observed to interfere with lignocellulose and protein peaks [15]. Increased salt content may additionally alter ionization probabilities during the ToF-SIMS measurement, potentially limiting the accuracy of the lignin and polysaccharide measurements with high salt content. However, one-way ANOVA statistical analysis of the data consistently showed that all chemical transformations observed by ToF-SIMS were significantly different at the 0.1 level due to treatment time, enzyme dose and the combination of the two. Additional file 10: Table S2. Peak assignments to xylose, selected xylo-oligosaccharides, and ABTS in reaction supernatants. Laccase and xylanase activity in reactions containing wood samples was evaluated by analysing reaction supernatants by mass spectrometry. Briefly, 10 μL of supernatant were injected into a Q-Exactive mass spectrometer (Thermo Scientific) and direct infusion mass spectra were collected in positive mode. Ions were collected in the range of 100-1000 m/z range at resolution 70,000. Spectral analyses were performed using Xcalibur 2.2 and SIEVE packages from Thermo Scientific. Calculated masses are shown for ABTS, xylose, and possible xylo-oligosaccharides released from xylanase treatment of acetylated glucuronoxylan; potential adducts are also indicated. Peak (m/z) values for xylose, xylobiose, and xylotriose were experimentally determined, as were potential interferences from laccase and xylanase samples. Empty cells indicate m/z values not detected in the direct infusion mass spectra.

## Abbreviations

ABTS: 2,2′-azino-bis(3-ethylbenzothiazoline-6-sulphonic acid)
MCR: multivariate curve resolution
NEXAFS: near edge X-ray absorption fine structure
PC: principal component
PCA: principal component analysis
STXM: scanning transmission X-ray microscopy
ToF-SIMS: time-of-flight secondary ion mass spectrometry
