# Supplementary material for: Direct and up-close views of plant cell walls show a leading role for lignin-modifying enzymes on ensuing xylanases
Source: Biotechnol Biofuels. 2014 Dec 31;7:496. doi: 10.1186/s13068-014-0176-9 (PMC4297432; doi:10.1186/s13068-014-0176-9)
Supplement: Additional file 1: Table S1. — Characteristic NEXAFS peaks for ABTS, lignin, polysaccharides, protein and resin [39-42]. [file 13068_2014_176_MOESM1_ESM.docx]

**Table S1. Peak assignments used to interpret NEXAFS spectra**

| **Transition and functional group** | **Published Peak Assignment (eV)** | **Our spectra** | **Peak maxima in reference spectra and (*PCA loadings*)** |
| --- | --- | --- | --- |
| 1s–π*,  aromatic C=C | 285.08-285.37 [20]  285.3 [41] | Lignin  ABTS  Resin  Protein | 285.3 (*285.45*)  285.2  285.06, 285.4  285.07, 285.4 |
| 1s–π*, aromatic C-N | 286.9 [41] | ABTS | 286.6 |
| 1s–π*, aromatic C-OH | 286.9-287.25 [20]  286.5-287.3 [39] | Lignin | 287.0 (*287.10 - 287.25*) |
| mixed π* CH_3_, CH_2_/3p Rydberg transitions | 287.6 [20] | ABTS  Resin | 287.6  287.5 shoulder |
| 1s–π*, C=O of ester group | 288.35-288.48 [20]  288.4- 288.7 [39] | Resin  Lignin (oxidized) | 288.4 eV, 288.5 shoulder  (*288.45-288.75*)  288.5 |
| 1s → π*,  -(NH2)-C=O | 288.3 [39] | Protein | 288.2 |
| 1s-3p/σ*, aliphatic C-OH | 289.10- 289.59 [20]  289.5 [39] | Poly-saccharides | Cellulose: 289.3  Xylan:289.2  (*289.35-289.50*) |
| 1s–4p/σ*, aliphatic C–H | 290.45 [39],  290.6 [40] | Poly-saccharides | Xylan: 290.6  Cellulose: 290.3  (*290.25-290.40*) |
| 1s–4p/σ*, Aliphatic C–C | 290.8–293 [42] | Poly-saccharides | (*290.85-291.15*) |
