# Supplementary figures and images for: Direct and up-close views of plant cell walls show a leading role for lignin-modifying enzymes on ensuing xylanases
Source: Biotechnol Biofuels. 2014 Dec 31;7:496. doi: 10.1186/s13068-014-0176-9 (PMC4297432; doi:10.1186/s13068-014-0176-9)

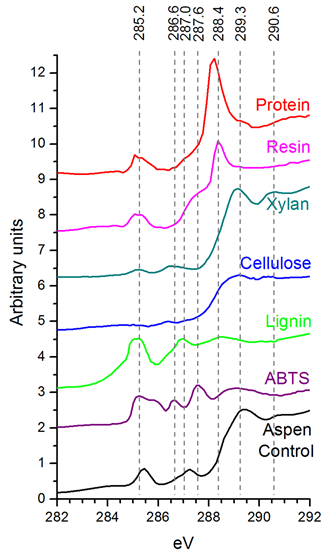

Supplement: Additional file 2: Figure S1. — Reference NEXAFS spectra. NEXAFS spectra of protein (bovine serum albumin), Spurr’s resin, xylan, cellulose, lignin, ABTS, and 1-day control aspen over the near-edge energy range of 282.0-292.0 eV are shown. Spectra for ABTS, Spurr’s resin, and aspen were collected in this current study; other spectra were previously collected by the Canadian Light Source (CLS). [file 13068_2014_176_MOESM2_ESM.tiff]

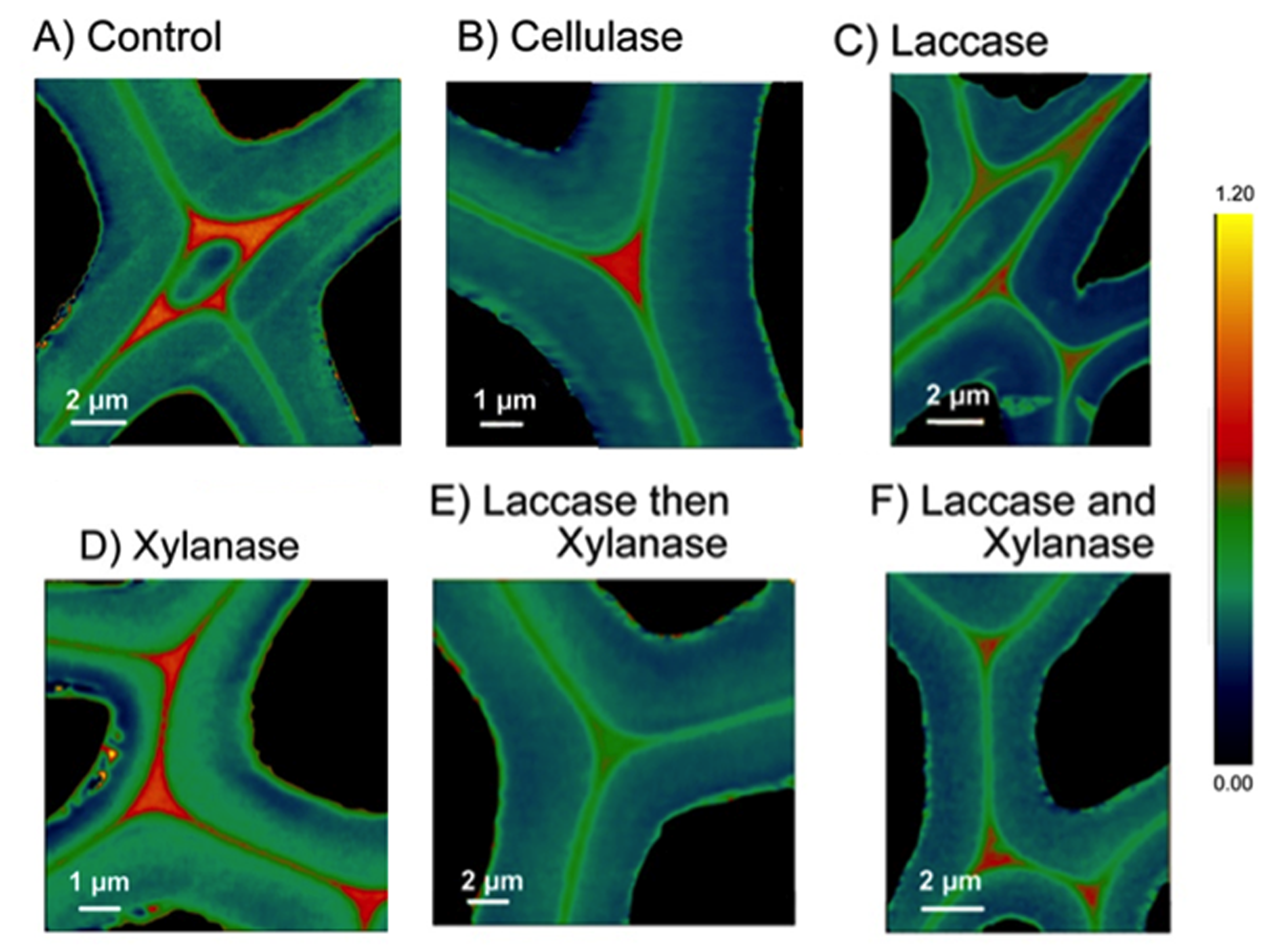

Supplement: Additional file 3: Figure S2. — STXM ratio images showing lignin to polysaccharides (as in Figure 1C) for untreated aspen (A), and for aspen treated with cellulase alone (B), laccase alone (C), xylanase alone (D), laccase followed by xylanase (E) and laccase co-incubated with xylanase (F). [file 13068_2014_176_MOESM3_ESM.zip › FigureS2.tiff]

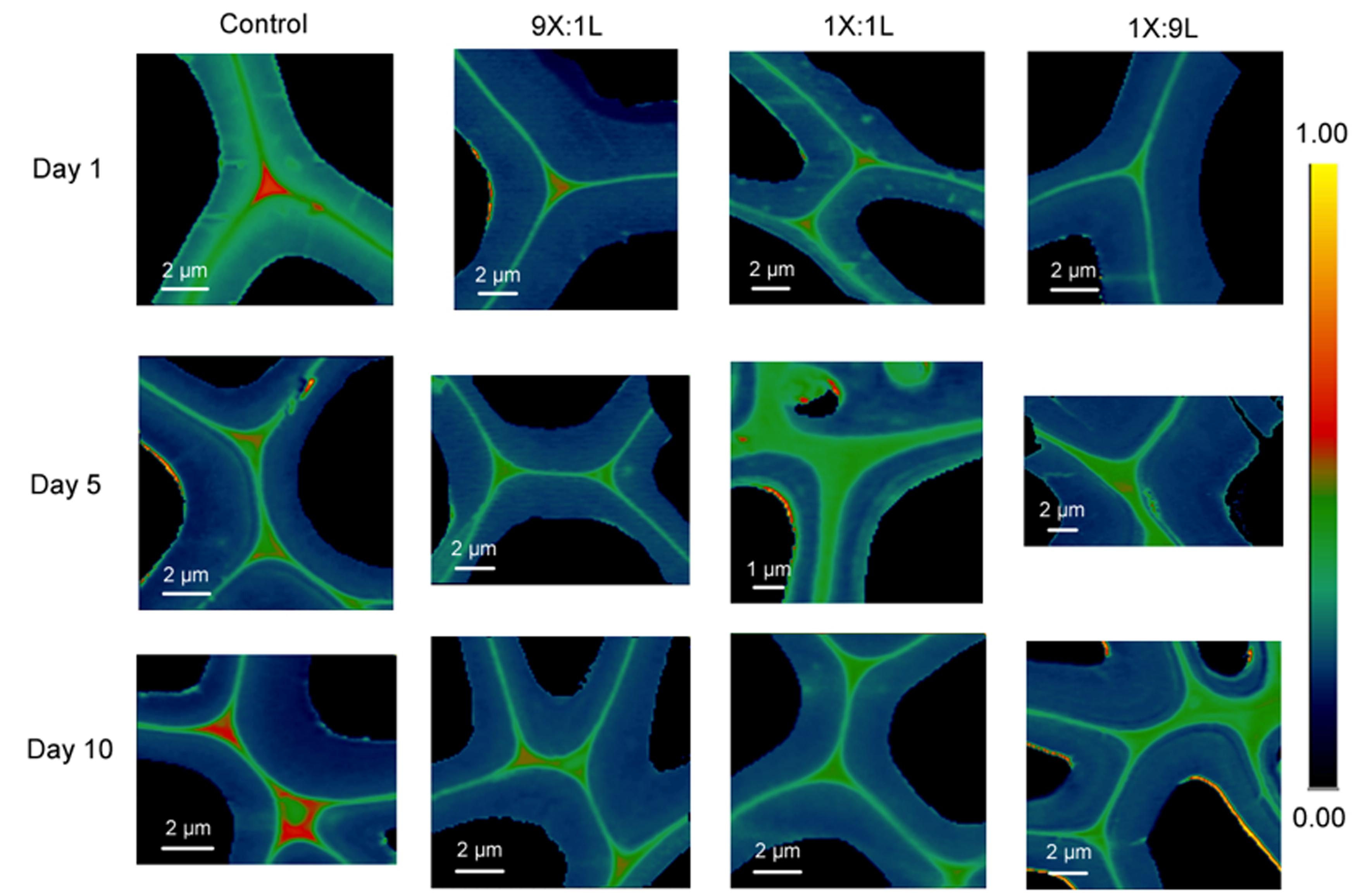

Supplement: Additional file 4: Figure S3. — STXM images showing lignin maps at 287.1 eV divided by polysaccharide maps at 289.3 eV for aspen treated with varying doses of xylanase and laccase for 1, 5, and 10 days. Images were prepared in the same way as Figure 1C. All images use the same colour scale, with warmer colours indicating more lignin-rich regions and cooler colours indicating more polysaccharide-rich regions. [file 13068_2014_176_MOESM4_ESM.zip › FigureS3.tiff]

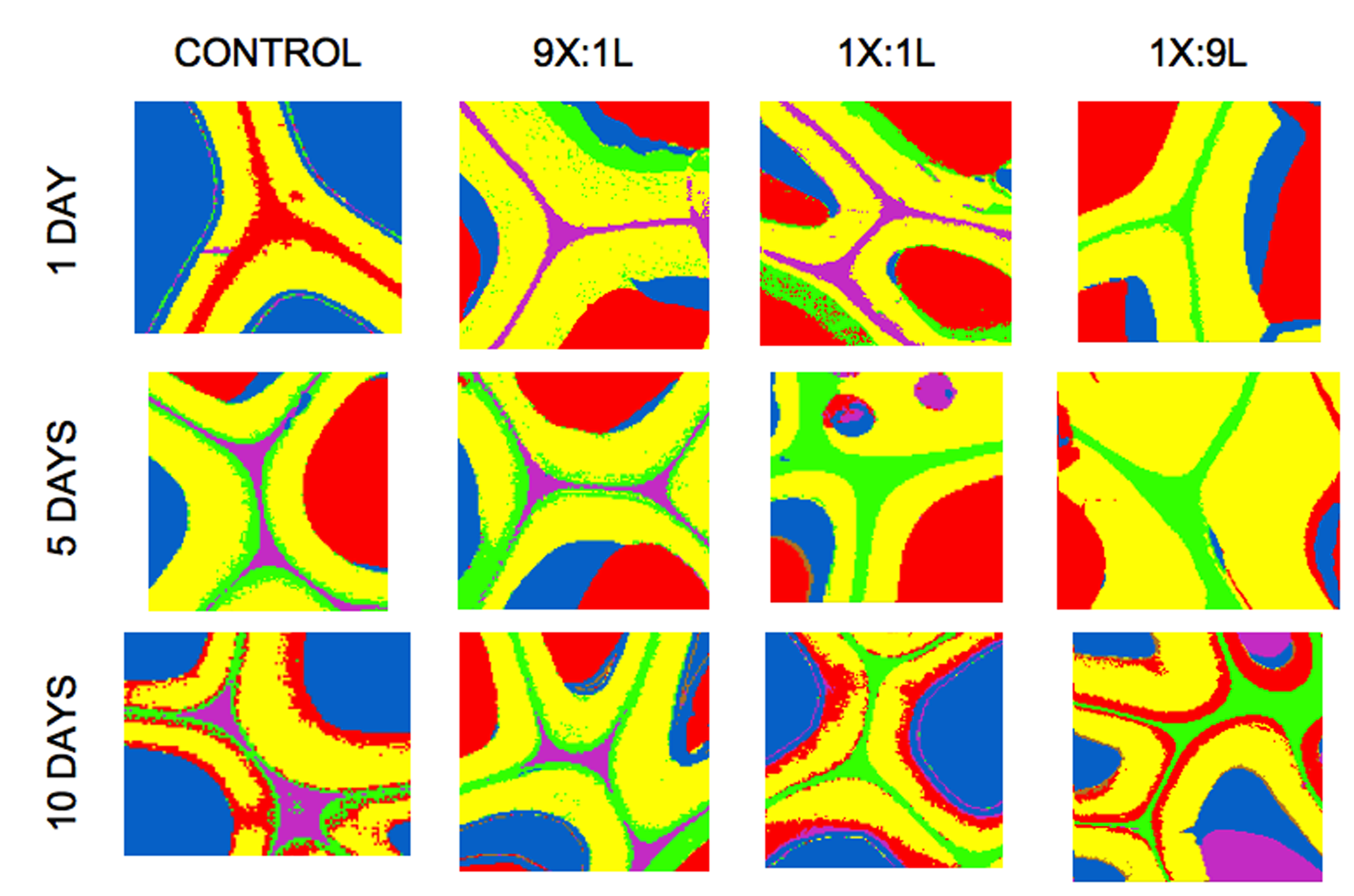

Supplement: Additional file 5: Figure S4. — Results of PCA-cluster analysis of STXM maps for samples treated with various doses of xylanase and laccase mixtures and ABTS for 1 day (top), 5 days (middle) and 10 days (bottom). Control samples were treated with ABTS only. The same colours within each image represent areas with the same chemistry. However, the colours between different images do not necessarily imply the same chemistry. [file 13068_2014_176_MOESM5_ESM.zip › FigureS4.tiff]

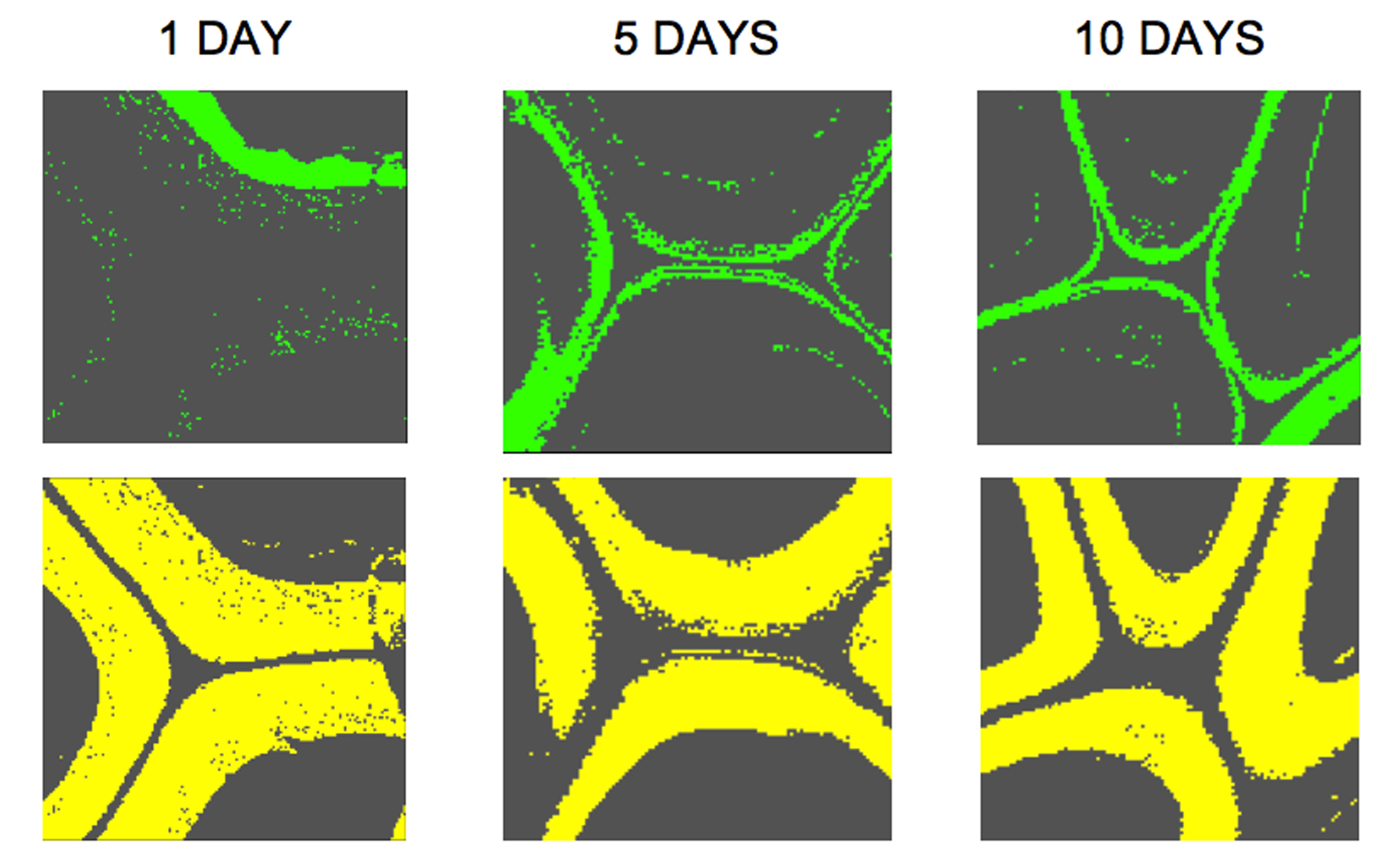

Supplement: Additional file 6: Figure S5. — Clusters within STXM maps showing S1/S3 layers (top, green) and S2 layers (bottom, yellow) for samples treated with high doses of xylanase (9X:1 L) for different periods of time (left: 1 day, middle: 5 days, right: 10 days). [file 13068_2014_176_MOESM6_ESM.zip › FigureS5.tiff]

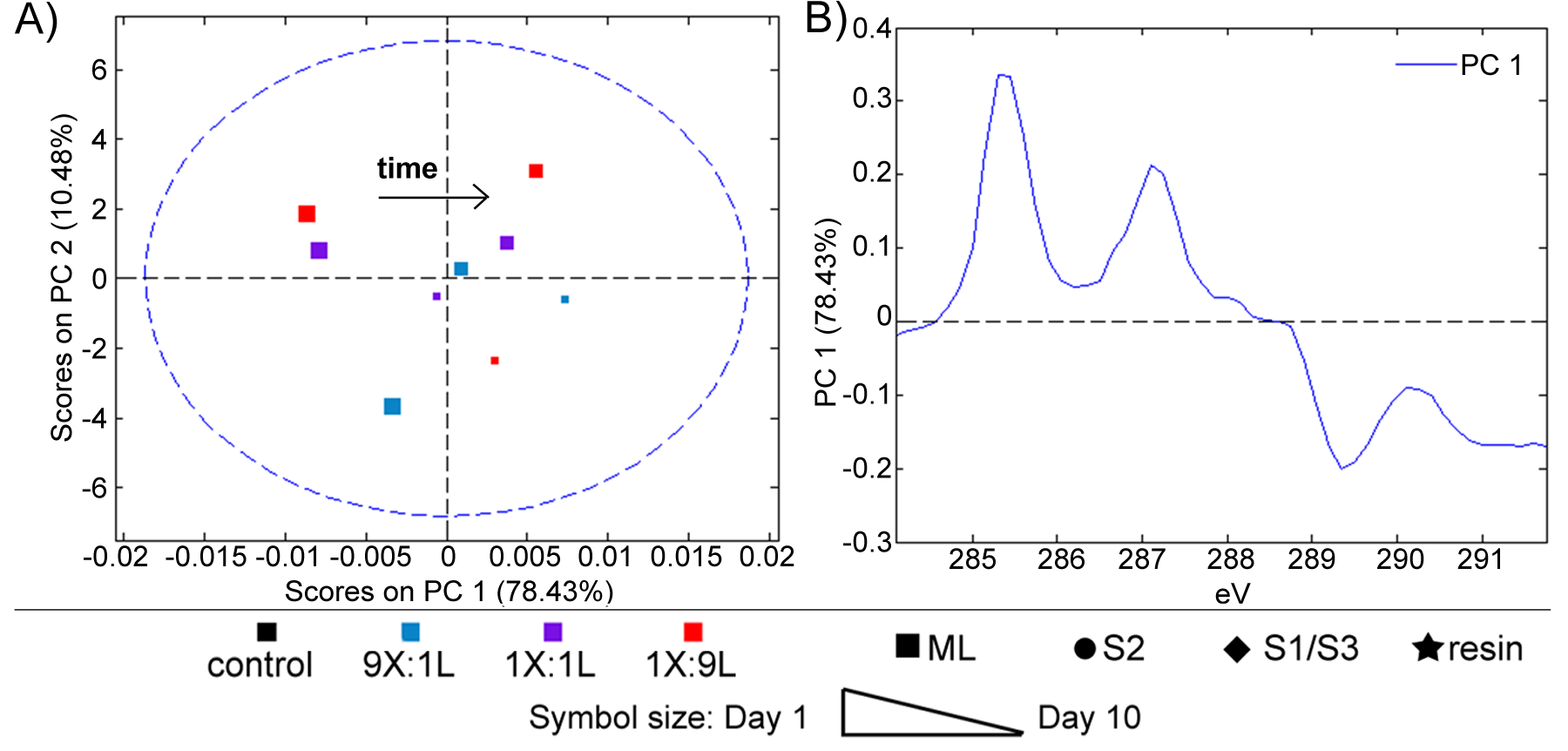

Supplement: Additional file 7: Figure S6. — PCA scores (A) and loadings (B) comparing STXM clusters from enzyme-treated middle lamella of the samples treated with varying xylanase and laccase ratios. [file 13068_2014_176_MOESM7_ESM.tiff]

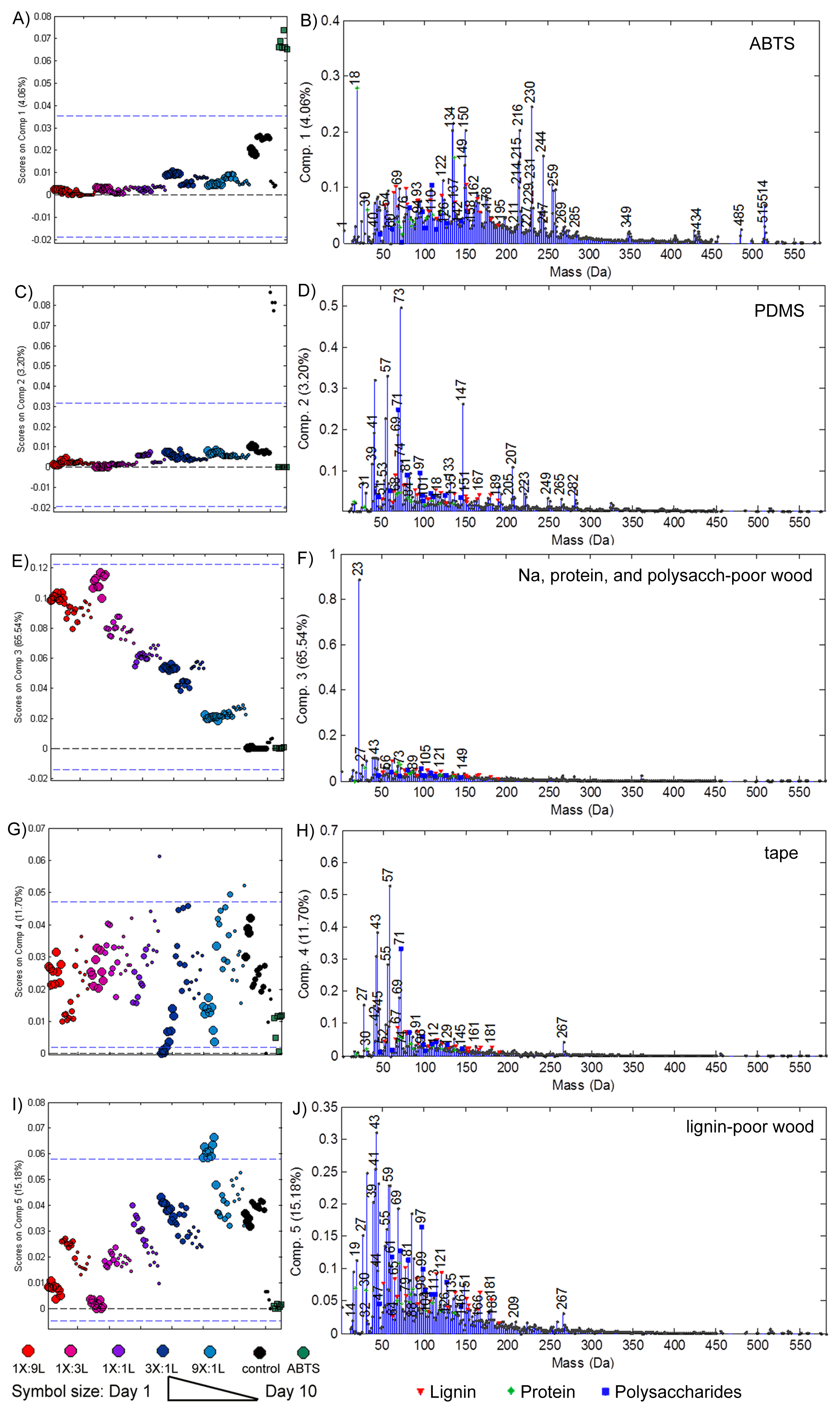

Supplement: Additional file 8: Figure S7. — MCR analysis of ToF-SIMS spectra showing scores (A, C, E, G, I) on the five component spectra (B, D, F, H, J). Component 1 (A, B) describes the ABTS mediator. Component 2 describes poly (dimethyl siloxane) (PDMS) contamination. Component 3 describes Na, protein, and lignin-rich wood. Component 4 describes the tape used to support the powdered wood. Component 5 describes lignin-depleted wood. [file 13068_2014_176_MOESM8_ESM.zip › FigureS7.tiff]

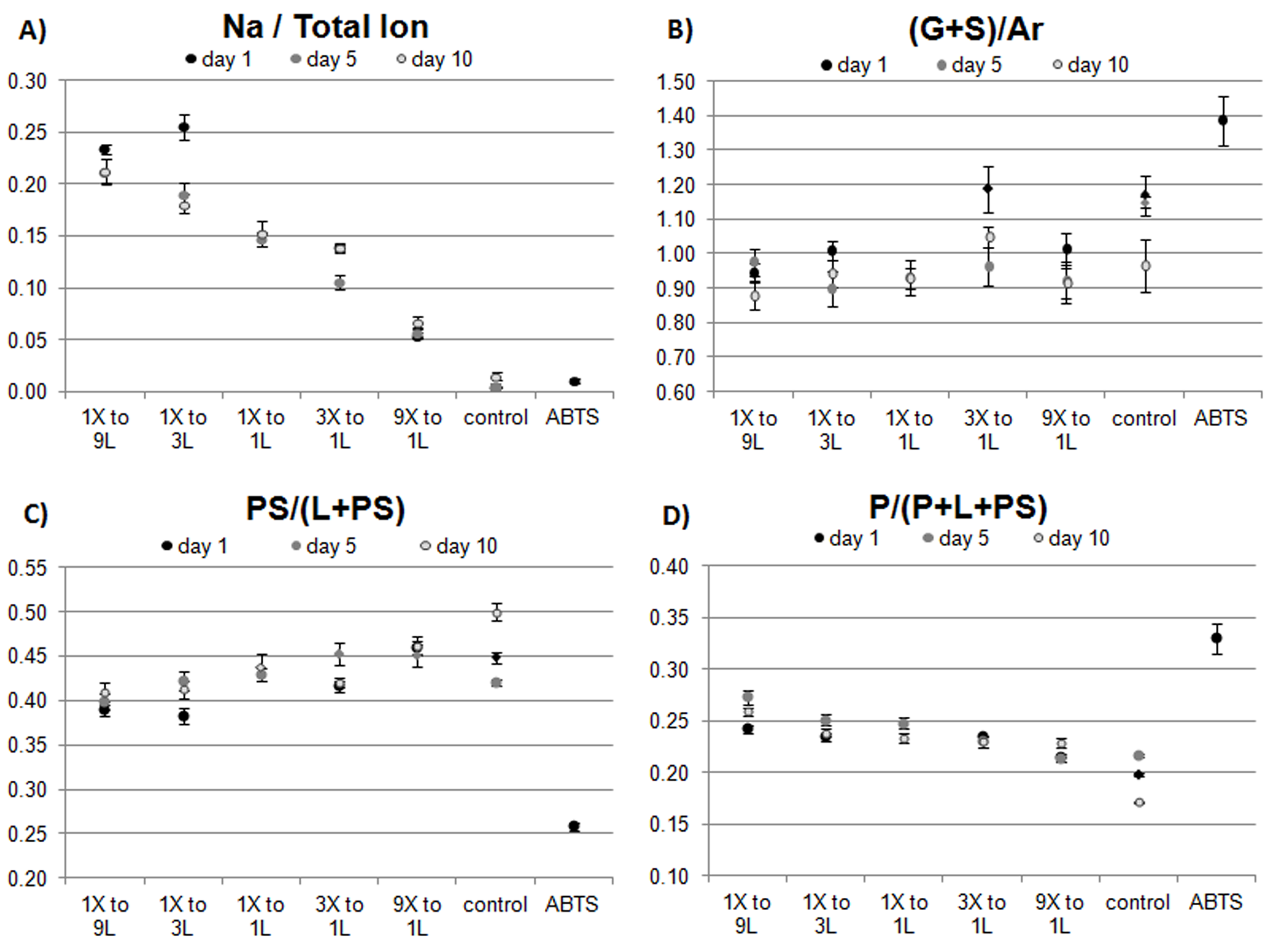

Supplement: Additional file 9: Figure S8. — Peak ratios from the raw ToF-SIMS spectra of the ground aspen samples, as described in Sodhi [13]: A) the proportion of the total spectrum comprised by the Na + peak at 23 Da; B) the lignin modification metric; C) the polysaccharide peak fraction and D) protein coverage. Peak ratios of pure ABTS deposited on a silicon wafer are also shown, to indicate whether ABTS coverage would increase or decrease the peak ratio. Notably, while Na + ions alone do not directly interfere with lignocellulose and protein peaks, at high levels of Na+, salt cluster ions have been observed to interfere with lignocellulose and protein peaks [15]. Increased salt content may additionally alter ionization probabilities during the ToF-SIMS measurement, potentially limiting the accuracy of the lignin and polysaccharide measurements with high salt content. However, one-way ANOVA statistical analysis of the data consistently showed that all chemical transformations observed by ToF-SIMS were significantly different at the 0.1 level due to treatment time, enzyme dose and the combination of the two. [file 13068_2014_176_MOESM9_ESM.tiff]
